# The Association Between Active Aging and Health-Related Quality of Life

**DOI:** 10.3390/geriatrics11030074

**Published:** 2026-06-17

**Authors:** Keon Woo, JungHoon Lee, YoonSoo Choy

**Affiliations:** 1 Graduate School of Transdisciplinary Health Sciences, Yonsei University, 50-1 Yonsei-ro, Seodaemun-gu, Seoul 03722, Republic of Korea; woopaper14@gmail.com (K.W.); leejung461@gmail.com (J.L.); 2 Department of Healthcare Management, Eulji University, 553 Sanseong-daero, Sujeong-gu, Seongnam-si 13135, Gyeonggi-do, Republic of Korea

**Keywords:** active aging, health-related quality of life, HINT-8, KNHANES

## Abstract

**Background and Objectives**: This study examined the association between active aging and health-related quality of life (HRQoL) in older Korean adults. Furthermore, it examined how socioeconomic factors were related to active aging and HRQoL. **Methods**: Data were analyzed using the 8th Korea National Health and Nutrition Examination Survey (KNHANES). This study used the Health-Related Quality of Life Instrument with Eight Items (HINT-8), a Korean-specific HRQoL instrument. Multivariable regression analyses were conducted to estimate the associations of overall active aging and its health, participation, and security domains with HRQoL. **Results**: The health domain showed the largest coefficient, and the security domain was also significantly associated with HRQoL. The participation domain showed a relatively limited and statistically non-significant association, which may partly reflect measurement constraints in the operationalization of participation. Older adults with lower educational levels and those residing in rural areas had lower levels of both active aging and HRQoL. **Conclusions**: Active aging was positively associated with HRQoL among older Korean adults. These findings highlight health as the domain most strongly associated with HRQoL and identify security as an additional relevant domain, while participation-related findings should be interpreted cautiously given the limited operationalization of participation and its statistically non-significant association. However, longitudinal or intervention studies are needed to examine temporal and causal relationships between active aging and HRQoL.

## 1. Introduction

Rapid population aging drives global demographic shifts and profoundly influences the broader social environment. Amidst these changes, the core challenge is no longer merely extending the biological lifespan but rather ensuring that this extended life is lived healthily and satisfyingly, with a specific focus on the quality of life of older adults. Health-related quality of life (HRQoL) is a multidimensional concept encompassing an individual’s perceived physical and mental health status as well as functional capabilities [1,2]. This concept is distinct from the medical state, defined simply by the absence of disease or frailty. Rather, it reflects the subjective satisfaction and sense of well-being experienced by older adults in performing daily activities and social roles [3]. Therefore, the ultimate goal of health policies for older adults is to enhance their perceived quality of life, enabling them to lead a satisfactory life.

In this context, the World Health Organization (WHO) has proposed “active aging” as a key policy framework for aging societies to improve their health-related quality of life [4,5]. Active aging is defined as the process of optimizing opportunities for health, participation, and security to enhance the quality of life as people age. This represents a paradigm shift from viewing older adults as passive recipients of care to recognizing them as active participants in society [4,6]. Active aging serves as a crucial practical strategy and process for achieving a health-related quality of life.

Previous studies have reported positive associations between active-aging-related factors and quality of life among older adults. Physical capability and regular activity have been linked to functional independence and life satisfaction [7,8]. Sustained social participation and leisure activities have also been associated with lower depressive symptoms, preserved cognitive function, and better quality of life [9,10]. In addition, safe housing environments and social protection systems have been discussed as environmental conditions related to psychological stability and active aging in later life [11].

However, despite the positive relationship between active-aging-related factors and quality of life, concerns have been raised regarding socioeconomic disparities in opportunities for active aging. Older adults with lower education or insufficient income may have lower health literacy, which can make it more difficult to access, understand, and use health information [12]. They may also have fewer opportunities for social participation, including lifelong learning [13]. Although these socioeconomic differences may be related to inequalities in active aging and quality of life, there remains a need for more detailed analysis of how these patterns appear across vulnerable groups [14].

Moreover, existing studies have limitations, as they often uncritically adopt theoretical frameworks established in Western sociocultural contexts or fail to comprehensively consider the multidimensional factors that determine quality of life [15]. In particular, most prior research has been limited to identifying fragmentary relationships between active aging and quality of life while failing to explain the complex effects of core active aging elements, such as health, participation, and security, on quality of life [14]. Furthermore, applying generic tools to measure quality of life across groups with diverse cultural and emotional backgrounds risks excluding the unique characteristics of these groups [1]. Therefore, it is uncommon and policy-relevant to conduct empirical research using optimized scales that encompass the multilayered attributes of active aging while precisely reflecting the sociocultural characteristics of the study population [2,16].

Previous studies have shown that active aging is associated with better quality of life among older adults and have identified health status, social participation, and environmental or economic security as relevant factors [14,16,17]. However, several gaps remain. First, many studies have examined selected components of active aging separately, making it difficult to compare the relative associations of the health, participation, and security domains within a single analytic framework [6,14]. Second, evidence from Korea remains limited regarding how active aging is associated with HRQoL when using a Korean-specific HRQoL instrument such as HINT-8 [1,2,16]. Third, although socioeconomic inequalities are central to aging policy, less is known about whether the association between active aging and HRQoL differs across socioeconomic groups among older Korean adults [12,13,14]. Addressing these gaps may help clarify which domains of active aging are more closely related to HRQoL and how these associations are distributed across social strata, thereby providing policy-relevant evidence for prioritizing multidimensional active aging strategies among older Korean adults.

Accordingly, this study aimed to examine the association between active aging and HRQoL among older Korean adults using nationally representative KNHANES data. Specifically, we first assessed the overall association between the AAI and HINT-8. Second, we examined the relative associations of the three core AAI domains—health, participation, and security—with HRQoL. Third, we explored whether these associations varied according to socioeconomic and health-related characteristics. Through these analyses, this study seeks to extend previous research by applying a multidimensional active aging framework to a Korean older adult population and by using HINT-8, a Korean-specific HRQoL instrument.

## 2. Methods

### 2.1. Population

In this study, we analyzed data from the 8th Korea National Health and Nutrition Examination Survey (KNHANES VIII, 2019–2021) to examine the association between active aging and health-related quality of life among older adults. The KNHANES is an annual, nationwide, cross-sectional survey based on a complex sample design that uses a multistage stratified probability sampling method to ensure representativeness of the Korean population. The primary objective of this survey was to generate reliable national statistics on the health status, health behaviors, dietary habits, and nutrient intake of the population, thereby providing essential evidence for the formulation and evaluation of public health policies.

The KNHANES comprises three main components—a health interview, medical examination, and nutrition survey—thereby serving as a robust and comprehensive data source for analyzing health-related outcomes in Korean adults. The EuroQol-5D (EQ-5D) has been used since 2005 to assess the quality of life of the population. Beginning with the 8th survey circle, the Health-Related Quality of Life Instrument with eight items (HINT-8) was administered in alternating years alongside the EQ-5D. Consequently, this study used data from 2019 and 2021, which included HINT-8, for its analysis.

**Figure 1:**
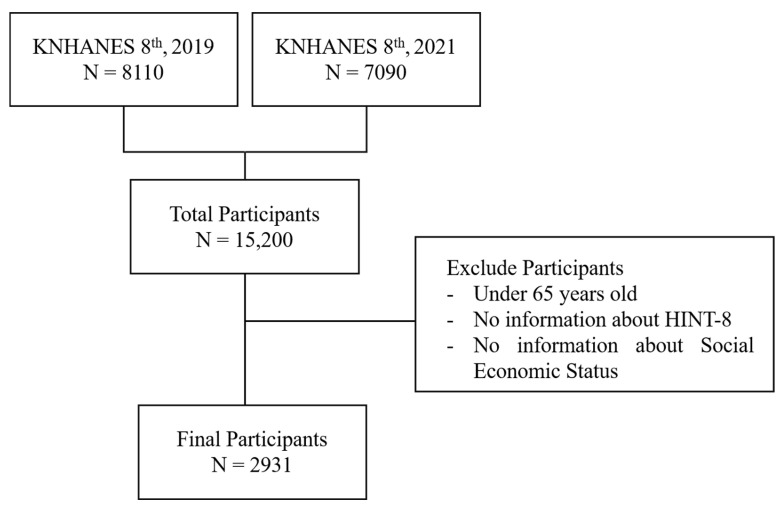
Flow Chart.

The selection process for study participants is shown in Figure 1. In the 8th KNHANES, 8110 participants were surveyed in 2019 and 7090 in 2021, yielding 15,200 individuals initially included in the study population. Among them, individuals who were not elderly, those without information on HINT-8, and those with missing information on AAI indicators or covariates were excluded using a complete-case approach. Consequently, 2931 participants were included in the final analysis. To assess potential selection differences due to missing information, we compared the characteristics of included participants and excluded older adults in Supplementary Table S1.

### 2.2. Independent Variable

Drawing on insights from a previous study, the Active Aging Index (AAI) was used to measure active aging in older adults [18]. AAI is an international metric designed to assess the extent to which the potential of older individuals is realized within society and the economy, effectively measuring the degree of active aging. The AAI comprises three domains: health, participation, and security [16]. Each domain was measured using the following variables. The selection of domain indicators was guided by the multidimensional AAI framework and by the availability of comparable variables in the KNHANES. Because KNHANES was not designed specifically to measure the full AAI, we used available indicators that most closely corresponded to the conceptual domains of health, participation, and security. Thus, the participation domain was operationalized using economic activity and household type as observable proxies for productive and social connectedness-related participation, whereas the security domain included indicators of economic, housing, insurance, and healthcare-access security.

The health domain included the number of chronic diseases, limitations in activities of daily living (ADL), perceived stress, subjective health status, and depressive symptoms. In the health domain, the number of chronic diseases was classified into three categories (0, 1, or ≥2) by summing the presence of nine primary chronic conditions, including hypertension, diabetes, dyslipidemia, myocardial infarction, stroke, arthritis, and asthma, based on physician diagnosis. Limitations in ADL, perceived stress, and depression were dichotomized into binary variables. Subjective health status was assessed using a 5-point scale (ranging from very poor to very good): good, moderate, and poor.

Economic activity status and household type were included in the participation domain. Economic activity was dichotomized based on employment status. Household type was categorized into elderly people living alone and those living with others, using generational composition information.

Finally, the security domain includes household income, homeownership, private health insurance enrollment, and unmet healthcare needs. Household income was categorized into quartiles. Homeownership and private health insurance enrollment were dichotomized based on their presence. Unmet healthcare needs were coded as yes or no in response to the question, “In the past year, have you been unable to receive medical care when needed?”

All variables were oriented such that higher values indicated a more positive social or health status. Negative factors, such as the number of chronic diseases and unmet healthcare needs, were reverse-coded to ensure consistency.

Subsequently, all indicators were normalized to range between 0 and 1 to standardize the scales across variables. Binary variables were utilized as is, whereas ordinal variables were transformed into a 0-1 range using their minimum and maximum values. For instance, the subjective health status (good, moderate, or poor) was normalized using the formula (x − 1)/(3 − 1).

The normalized indicators were averaged within each domain to derive the health, participation, and security domain scores. Specifically, the health domain score was calculated as the mean of the normalized scores for chronic disease, ADL limitations, perceived stress, subjective health status, and depressive symptoms. The participation-domain score was determined by averaging the economic activity status and household type. Security-domain scores were computed from the means of household income, homeownership, private insurance enrollment, and unmet medical needs.

Following previous AAI-based research, normalized indicators were averaged within each domain, and the three domain scores were averaged using equal weightings to generate an individual’s integrated AAI [16]. This equal-weighting approach was used because externally validated weights for the relative importance of each AAI component in the Korean older adult population were not available, and because it provides a transparent and reproducible method for summarizing multidimensional indicators. The AAI ranged from 0 to 1, with higher values indicating greater active aging.

### 2.3. Dependent Variable

The dependent variable in this study was the HINT-8. HINT-8 is a Korean-specific HRQoL instrument developed to capture multiple dimensions of health, including physical, social, mental, and positive health domains. Previous studies have suggested that HINT-8 may reduce concerns related to the ceiling effect observed in EQ-5D and may better reflect HRQoL in the Korean population. Its validity is supported by its extensive use in various studies [1,2]. HINT-8 comprises eight items that encompass the physical, social, mental, and positive dimensions of health. It measures the respondents’ status over the past week concerning climbing, pain, vitality, work, depression, memory, sleep, and happiness. Disutility weights were applied to each of the four response options across the eight items, and the resulting decrements were summed. The scores ranged from 0.132 to 1, with higher scores indicating better health-related quality of life.

### 2.4. Covariates

This study incorporates several covariates, primarily encompassing socioeconomic status and health-related factors. Sex was categorized as male or female. The residents were divided into urban and rural areas. Age groups were established as 65–74 years and ≥75 years. Education level was classified as high, middle, or low. The smoking status included current smokers, former smokers, and nonsmokers. Drinking status was dichotomized. Body mass index (BMI) was categorized as underweight, normal weight, overweight, or obese.

### 2.5. Statistical Analysis

Based on the conceptual framework, this study used the following analysis to examine the relationships between the variables. Given that the KNHANES uses a complex sample design, all data analyses incorporate methods for complex survey data, accounting for sampling weights and stratification and cluster variables. First, descriptive statistics were used to examine the shape of the data distribution. After controlling for covariates, the association between the AAI and HINT-8 in older adults was analyzed using multiple linear regression. For interpretability, the coefficients for the overall AAI and each AAI domain represent a 0.1-unit increase in the corresponding score. Sensitivity analysis was then conducted by disaggregating the three domains of the AAI to examine the independent relationship between each domain and the HINT-8. Subgroup analyses were conducted to examine whether the association between active aging and HINT-8 was consistent across key sociodemographic and health-related subgroups. In addition to estimating subgroup-specific coefficients, we fitted survey-weighted regression models with interaction terms between AAI and each subgroup variable. The p-values for interactions were reported to formally assess whether the AAI-HINT-8 association differed across subgroup categories.

In addition, following the recommendation to examine social strata using a covariance structure framework, we conducted a supplementary multi-group path analysis by educational level. Educational level was used as the primary indicator of socioeconomic stratum because it was specified as a key socioeconomic characteristic and was not included as a component of the AAI. The AAI-HINT-8 path was freely estimated across educational strata in the unconstrained model and was constrained to be equal across strata in the constrained model. The results are presented in Supplementary Table S4.

To empirically examine ceiling effects, we conducted a descriptive comparison of HINT-8 and EQ-5D scores in the 2019 subsample, where both measures were available. We calculated the mean, standard deviation, and ceiling proportion for each measure and visually compared their score distributions using histograms. The ceiling for HINT-8 was defined as the maximum observed HINT-8 score in the 2019 subsample, whereas the ceiling for EQ-5D was defined as an EQ-5D index score of 1 (Supplementary Table S2 and Supplementary Figure S1).

All statistical analyses were performed using SAS (version 9.4; SAS Institute Inc., Cary, NC, USA), and a two-sided *p* < 0.05 was considered statistically significant.

## 3. Results

Table 1 presents the descriptive statistics of the study population. In total, 2931 older adults were included in the analysis. The mean AAI score was 0.63 (SD = 0.18), and the mean HINT-8 score was 0.77 (SD = 0.11).

Regarding socioeconomic characteristics, females made up 55.6% (*n* = 1631) of the sample, whereas males accounted for 44.4% (*n* = 1300). Of the participants, 69.6% lived in urban areas (*n* = 2040), and 30.4% (*n* = 891) lived in rural areas. In terms of age distribution, 61.8% (*n* = 1810) were aged 65–74 years, and 38.2% (*n* = 1121) were 75 years or older. Of the participants, 52.3% (*n* = 1533) had low educational levels, 37.0% (*n* = 1084) moderate educational levels, and 10.7% (*n* = 314) high educational levels.

Regarding health behaviors, 61.7% (*n* = 1808) were nonsmokers, 28.9% (*n* = 846) were former smokers, and 9.5% (*n* = 277) were current smokers. Nearly half of the participants reported consuming alcohol (48.1%, *n* = 1411), whereas 51.9% (*n* = 1520) did not consume alcohol. The BMI distribution showed that 36.5% (*n* = 1071) were obese, 26.8% (*n* = 785) were overweight, 33.6% (*n* = 985) were normal weight, and 3.1% (*n* = 90) were underweight.

Table 2 presents the results of a multiple linear regression analysis examining the association between AAI and HINT-8 scores in older adults after controlling for socioeconomic and health-related covariates. The analysis showed that AAI was positively associated with the HINT-8. Specifically, a 0.1-unit higher AAI score was associated with a 0.220-point higher HINT-8 score (β = 0.220, SE = 0.013, *p* < 0.0001).

Among the covariates, sex was significantly associated with the HINT-8 scores. Females exhibited lower HINT-8 scores than males (β = −0.030, SE = 0.006, *p* < 0.0001). Educational level was another significant factor. Participants with lower educational levels demonstrated significantly lower HINT-8 scores than those with higher educational levels (β = −0.033, SE = 0.007, *p* < 0.0001). While the group with middle education levels tended to have lower scores (β = −0.006, SE = 0.006), this did not reach statistical significance (*p* = 0.291). Current smoking status showed a statistically significant negative association with HINT-8 scores (β = −0.018, SE = 0.009, *p* = 0.039), whereas former smokers exhibited a borderline trend that did not reach statistical significance (*p* = 0.058).

Other covariates, including residence, age group, drinking status, and BMI, did not demonstrate statistically significant associations with the HINT-8 in this model.

Table 3 presents the results of simultaneously entering the three AAI domains. The coefficients represent differences in HINT-8 per 0.1-unit higher domain score. The health domain showed the largest coefficient and was positively associated with HINT-8 (β = 0.260, SE = 0.010, *p* < 0.0001). The security domain also showed a statistically significant positive association with HINT-8, although the magnitude was smaller than that of the health domain (β = 0.052, SE = 0.009, *p* < 0.0001). The participation domain showed a small association, but it was not statistically significant (β = 0.011, SE = 0.006, *p* = 0.067).

Table 4 presents the subgroup-specific associations between AAI and HINT-8, along with p-values for interaction. AAI was positively associated with HINT-8 across all examined subgroups. The regression coefficients ranged from 0.019 to 0.028 per 0.1-unit increase in AAI, and all subgroup-specific associations were statistically significant (all *p* < 0.0001).

The *p*-for-interaction values were 0.689 for sex, 0.900 for residence, 0.168 for age group, 0.090 for education level, 0.059 for smoking status, 0.950 for drinking status, and 0.260 for BMI. None of the results of these interaction tests reached statistical significance. These results indicate that, although subgroup-specific point estimates varied numerically, there was no clear statistical evidence that the AAI-HINT-8 association differed by sex, residence, age group, education level, smoking status, drinking status, or BMI.

## 4. Discussion

This study examined the association between active aging and HRQoL among older Korean adults using data from the 8th Korea National Health and Nutrition Examination Survey (KNHANES). The overall AAI was positively associated with HINT-8, and the three AAI domains showed different patterns of association with HRQoL. Among the domains, health showed the largest coefficient; security was also positively and significantly associated with HRQoL, and participation showed a relatively limited and statistically non-significant association that may partly reflect measurement constraints. In addition, older adults with lower educational levels or those residing in rural areas tended to have lower levels of both active aging and HRQoL. These findings should be interpreted as cross-sectional associations rather than causal effects.

Although the association between AAI and HINT-8 was statistically significant, its practical interpretation requires caution. The coefficient of 0.220 indicates that a 0.1-unit higher AAI score was associated with a 0.220-point higher HINT-8 score. Given that the HINT-8 score ranged from 0.132 to 1 in this study, this association may be meaningful in magnitude. However, because the analysis was cross-sectional, this coefficient should not be interpreted as a clinical effect or as evidence that increasing active aging would directly improve HRQoL. Rather, it indicates a cross-sectional difference in HRQoL associated with differences in active aging levels.

This study used HINT-8 as a Korean-specific HRQoL measure designed to capture multiple dimensions of health among older adults. Although previous studies have raised concerns that EQ-5D may show ceiling effects in relatively healthy populations, our additional comparison in the 2019 subsample also suggested that HINT-8 was less concentrated at the maximum score than EQ-5D. However, because this comparison was limited to the 2019 subsample, the use of HINT-8 should be interpreted as a methodological choice supported by prior validation studies and supplementary descriptive evidence, rather than as definitive evidence that HINT-8 fully overcomes the ceiling effect of EQ-5D.

The relatively larger coefficient for the health domain is consistent with previous studies emphasizing physical function, chronic disease management, and independence in later life as important correlates of quality of life [8,19,20,21]. Related evidence has also linked positive aging expectations, physical activity, depressive symptoms, and other health indicators with well-being or life satisfaction in middle-aged and older adults [22,23]. In this study, older adults with more favorable health-domain scores tended to report higher HINT-8 scores. This pattern may reflect the close conceptual relationship between health status, functional capacity, and perceived quality of life in older age. However, because the data are cross-sectional, this finding does not establish that better health-domain status leads to higher HRQoL. It is also possible that older adults with better HRQoL are more likely to maintain health-related behaviors and functional independence.

The relatively small and statistically non-significant coefficient for the participation domain should be interpreted cautiously. In this study, the participation domain was measured using only economic activity and household type, which may not fully capture broader dimensions of social participation, such as volunteering, community engagement, lifelong learning, perceived meaningfulness, or quality of relationships. Previous studies have suggested that meaningful participation, relationship quality, activity patterns, and experiences of discrimination may be relevant to well-being and quality of life in older adults [17,24,25,26,27]. Studies of active-maturity groups and peer-volunteering interventions have also indicated the potential relevance of structured social engagement in later life [28,29]. However, these qualitative and contextual aspects of participation could not be directly examined in the present study. Therefore, the limited association observed for participation may partly reflect measurement constraints rather than the absence of a relationship between social participation and HRQoL. In addition, unmeasured psychosocial factors, such as social support, loneliness, perceived usefulness, or depressive symptoms beyond the measured indicators, may have influenced the observed association. Future studies using more granular measures of participation are needed to clarify this association.

The security domain also warrants careful interpretation. Although its coefficient was smaller than that of the health domain, security remained positively and statistically significantly associated with HRQoL. This suggests that economic, housing, insurance, and healthcare-access resources may be relevant to HRQoL among older adults, even after accounting for the health and participation domains. In particular, the security domain may point to policy levers that differ from those of the health domain, such as reducing unmet healthcare needs, improving access to care, and strengthening social and material resources in later life. At the same time, the composition of the security domain should be considered when interpreting this result. Because unmet healthcare needs may conceptually overlap with healthcare access and health status, the security coefficient should not be interpreted as reflecting material or environmental security alone. Although the multicollinearity diagnostics did not indicate substantial statistical overlap among the AAI domains, conceptual overlap remains possible and should be considered when interpreting the relative magnitude of the domain-specific coefficients.

The observed differences by educational level suggest that active aging and HRQoL are unequally distributed across socioeconomic groups. Lower educational attainment may be related to lower health literacy, which can make it more difficult for older adults to access, understand, and use health information [12]. Older adults with fewer socioeconomic resources may also have fewer opportunities for social participation, including lifelong learning [13]. These factors may partly explain why older adults with lower educational levels had lower AAI and HINT-8 scores in this study. In addition, previous studies have emphasized the role of older adult education and health literacy in supporting active aging [12,30]. However, because of the cross-sectional design, these findings should not be interpreted as evidence that socioeconomic disadvantage causes lower active aging or HRQoL. Rather, they suggest the need for future longitudinal research to examine how socioeconomic resources, active aging, and HRQoL are connected over time.

Several limitations should be noted. First, because this study used a cross-sectional design, causal relationships and temporal ordering between active aging and HRQoL could not be established. Although higher AAI scores were associated with higher HINT-8 scores, it remains unclear whether active aging contributes to better HRQoL, whether older adults with better HRQoL are more likely to engage in active aging, or whether both are influenced by unmeasured factors. Therefore, the findings should be interpreted as associations. Longitudinal studies are needed to examine temporal relationships, and intervention studies are required to determine whether improvements in active aging domains lead to subsequent changes in HRQoL.

Second, the operationalization of the AAI domains was constrained by the variables available in KNHANES. The participation domain did not capture broader qualitative and community-based forms of participation, and unmet healthcare needs in the security domain may partly overlap with health status or healthcare access. These measurement choices may have attenuated the estimated association for participation and may have influenced the apparent contribution of the security domain. In addition, the AAI was constructed using an equal-weighting approach. Although this approach is transparent and consistent with previous AAI-based research, it implicitly assumes that each component contributes equally to active aging. Therefore, the domain and overall AAI scores should be interpreted as pragmatic summary measures rather than as empirically weighted estimates of the relative importance of each component.

Taken together, these findings may inform hypotheses and potential priorities for aging-related policy. The strong association between the health domain and HRQoL suggests that preventive health management, chronic disease self-management, and maintenance of physical function remain important areas for policy attention [20,31]. The findings also suggest that security should not be overlooked, whereas the participation-related findings should be interpreted cautiously because the participation domain was measured using a limited set of indicators, and its association with HRQoL was not statistically significant. In particular, policy efforts targeting older adults with lower educational levels or those living in rural areas may be relevant for reducing disparities in active aging resources and HRQoL, but the effectiveness of such interventions should be evaluated in future studies.

## Figures and Tables

**Table 1:** General Characteristics of study participants.

		N/Mean	%/SD
Total		2931	100.0
AAI		0.63	0.18
HINT-8		0.77	0.11
Sex			
	Male	1300	44.4
	Female	1631	55.6
Residence			
	Urban	2040	69.6
	Rural	891	30.4
Age group			
	65-74	1810	61.8
	≥75	1121	38.2
Education level			
	High	314	10.7
	Middle	1084	37.0
	Low	1533	52.3
Smoke			
	None	1808	61.7
	Former	846	28.9
	Current	277	9.5
Drink			
	Yes	1411	48.1
	No	1520	51.9
BMI			
	Underweight	90	3.1
	Normal	985	33.6
	Overweight	785	26.8
	Obesity	1071	36.5

Note. AAI, Active Aging Index; HINT-8, Health-Related Quality of Life Instrument with Eight Items; BMI, Body Mass Index; SD, standard deviation.

**Table 2:** Multiple linear regression analysis of factors associated with HINT-8.

		β	SE	*p* Value
AAI		0.220	0.013	<0.0001
Sex				
	Male	ref		
	Female	−0.030	0.006	<0.0001
Residence				
	Urban	ref		
	Rural	0.002	0.004	0.661
Age group				
	65–74	ref		
	≥75	−0.003	0.004	0.502
Education level				
	High	ref		
	Middle	−0.006	0.006	0.291
	Low	−0.033	0.007	<0.0001
Smoke				
	None	ref		
	Former	−0.011	0.006	0.058
	Current	−0.018	0.009	0.039
Drink				
	Yes	0.008	0.004	0.068
	No	ref		
BMI				
	Underweight	−0.015	0.013	0.259
	Normal	ref		
	Overweight	−0.001	0.005	0.937
	Obesity	−0.007	0.005	0.108

Note. AAI, Active Aging Index; HINT-8, Health-Related Quality of Life Instrument with Eight Items; BMI, Body Mass Index; SE, standard error. The coefficient for AAI represents the difference in HINT-8 per 0.1-unit higher AAI score.

**Table 3:** Association between HINT-8 and AAI domains.

Domain	β	SE	*p* Value
Health	0.260	0.010	<0.0001
Participation	0.011	0.006	0.067
Security	0.052	0.009	<0.0001

Note. AAI, Active Aging Index; HINT-8, Health-Related Quality of Life Instrument with Eight Items; SE, standard error. Coefficients represent differences in HINT-8 per 0.1-unit higher domain score. Models adjusted for sex, residence, age group, education, smoking, drinking, and BMI.

**Table 4:** Subgroup association between AAI and HINT-8.

	β	SE	95% CI	*p*-Value	*p*-for-Interaction
Sex					0.689
Male	0.021	0.002	0.018 to 0.024	<0.0001	
Female	0.023	0.002	0.020 to 0.026	<0.0001	
Residence					0.900
Urban	0.021	0.001	0.019 to 0.024	<0.0001	
Rural	0.026	0.002	0.022 to 0.030	<0.0001	
Age group					0.168
65–74	0.02	0.002	0.017 to 0.023	<0.0001	
≥75	0.025	0.002	0.022 to 0.028	<0.0001	
Education level					0.090
High	0.019	0.003	0.014 to 0.025	<0.0001	
Middle	0.021	0.002	0.018 to 0.024	<0.0001	
Low	0.026	0.002	0.023 to 0.029	<0.0001	
Smoke					0.059
None	0.022	0.001	0.020 to 0.025	<0.0001	
Former	0.021	0.002	0.018 to 0.025	<0.0001	
Current	0.024	0.004	0.017 to 0.031	<0.0001	
Drink					0.950
Yes	0.021	0.002	0.018 to 0.024	<0.0001	
No	0.024	0.002	0.021 to 0.027	<0.0001	
BMI					0.260
Underweight	0.028	0.006	0.016 to 0.040	<0.0001	
Normal	0.023	0.002	0.020 to 0.026	<0.0001	
Overweight	0.021	0.002	0.018 to 0.025	<0.0001	
Obesity	0.022	0.002	0.019 to 0.025	<0.0001	

Note. β represents the regression coefficient for HINT-8 per 0.1-unit increase in AAI within each subgroup. *p*-for-interaction was obtained from a single survey-weighted regression model including the interaction term between AAI and each subgroup variable. For subgroup variables with more than two categories, *p*-for-interaction represents the overall test of the interaction terms. AAI, Active Aging Index; HINT-8, Health-Related Quality of Life Instrument with Eight Items; SE, standard error; CI, confidence interval; BMI, body mass index.

## Data Availability

This study uses the Korea National Health and Nutrition Examination Survey. The data is available for statistical analysis and can be accessed through the Korea Disease Control and Prevention Agency’s Korea National Health and Nutrition Examination Survey portal.

## Supplementary Materials

The following supporting information can be downloaded at: https://www.mdpi.com/article/10.3390/geriatrics11030074/s1, Table S1: Characteristics of Included and Excluded Older Adults; Table S2: Descriptive Statistics and Ceiling Proportions of HINT-8 and EQ-5D in the 2019 Subsample; Figure S1: Distribution of HINT-8 and EQ-5D Scores in the 2019 Subsample; Table S3: Multicollinearity Diagnostics for the Main and Domain-Specific Models; Table S4: Multi-group path analysis of the association between AAI and HINT-8 by educational level.

## Abbreviations

HRQoL	Health-Related Quality of Life
KNHANES	Korea National Health and Nutrition Examination Survey
EQ-5D	The EuroQol-5D
HINT-8	Health-Related Quality of Life Instrument with eight items
WHO	World Health Organization
AAI	Active Aging Index
ADL	Activities of Daily Living
BMI	Body Mass Index
SE	Standard Error
SD	Standard Deviation

## References

[1] Hong G.-U., Koo B.S., Kim M.-J., Sim W.-J., Lee A.-Y., An J.-E., Yu S.-Y., Lee S.Y., Lee S.H. (2025). Validation of the health-related quality of life instrument with 8 items for assessing health-related quality of life in patients with oropharyngeal cancer: A comparison with the EQ-5D-5L. Health Qual. Life Outcomes.

[2] Kim J., Lee H.-J., Jo M.-W. (2022). Health-related quality of life instrument with 8 items for use in patients with type 2 diabetes mellitus: A validation study in Korea. J. Prev. Med. Public Health.

[3] Hyun E. (2020). The impact of active aging of the elderly on life satisfaction in the aged society. J. Humanit. Soc. Sci..

[4] Fernández-Ballesteros R., Robine J.M., Walker A., Kalache A. (2013). Active aging: A global goal. Curr. Gerontol. Geriatr. Res..

[5] López-López R., Sánchez M. (2020). The institutional active aging paradigm in Europe (2002–2015). Gerontologist.

[6] Bélanger E., Ahmed T., Filiatrault J., Yu H.-T., Zunzunegui M.V. (2017). An empirical comparison of different models of active aging in Canada: The international mobility in aging study. Gerontologist.

[7] An H.-Y., Chen W., Wang C.-W., Yang H.-F., Huang W.-T., Fan S.-Y. (2020). The relationships between physical activity and life satisfaction and happiness among young, middle-aged, and older adults. Int. J. Environ. Res. Public Health.

[8] Sulandari S., Coats R.O., Miller A., Hodkinson A., Johnson J. (2024). A systematic review and meta-analysis of the association between physical capability, social support, loneliness, depression, anxiety, and life satisfaction in older adults. Gerontologist.

[9] Flores Tena M.J., Deocano-Ruiz Y., Llamas-Salguero F., Jiménez Morales J.J. (2023). Active aging with leisure and free time activities for a better quality of life. Retos.

[10] Lv R., Yang L., Li J., Wei X., Ren Y., Wang W., Hou J., Fang X. (2024). Relationship between social participation and life satisfaction in community-dwelling older adults: Multiple mediating roles of depression and cognitive function. Arch. Gerontol. Geriatr..

[11] da Silva Sousa N.F., de Azevedo Barros M.B. (2020). Level of active aging: Influence of environmental, social and health-related factors. Arch. Gerontol. Geriatr..

[12] Eronen J., Paakkari L., Portegijs E., Saajanaho M., Rantanen T. (2021). Health literacy supports active aging. Prev. Med..

[13] Morris-Foster J.M. (2024). The influence of lifelong learning on life satisfaction and successful aging in older adults: A narrative literature review. J. Gerontol. Nurs..

[14] Marzo R.R., Khanal P., Shrestha S., Mohan D., Myint P.K., Su T.T. (2023). Determinants of active aging and quality of life among older adults: Systematic review. Front. Public Health.

[15] Shum T.C.T. (2023). Quality of life of South Asian older adults in Hong Kong: Policy implications for a multicultural active aging framework. Gerontol. Geriatr. Med..

[16] Eum M., Kim H. (2021). Relationship between active aging and quality of life in middle-aged and older Koreans: Analysis of the 2013–2018 KNHANES. Healthcare.

[17] Marsillas S., De Donder L., Kardol T., van Regenmortel S., Dury S., Brosens D., Smetcoren A.-S., Braña T., Varela J. (2017). Does active ageing contribute to life satisfaction for older people? Testing a new model of active ageing. Eur. J. Ageing.

[18] Seo J., Joo K., Li Y., Kim N., Oh E., Gansukh L., Song R. (2025). Healthy aging in frail older adults: Active aging project of a national survey. Int. J. Nurs. Stud. Adv..

[19] Paúl C., Teixeira L., Ribeiro O. (2017). Active aging in very old age and the relevance of psychological aspects. Front. Med..

[20] Canhão H., Branco J.C., Liotta G. (2018). Editorial: Active aging and disease management. Front. Med..

[21] Boccaccio D.E., Cenzer I., Covinsky K.E. (2021). Life satisfaction among older adults with impairment in activities of daily living. Age Ageing.

[22] Andrews R.M., Tan E.J., Varma V.R., Rebok G.W., Romani W.A., Seeman T.E., Gruenewald T.L., Tanner E.K., Carlson M.C. (2017). Positive aging expectations are associated with physical activity among urban-dwelling older adults. Gerontologist.

[23] An L., Ma L., Xu N., Yu B. (2023). Life satisfaction, depressive symptoms, and blood pressure in the middle-aged and older Chinese population. J. Psychosom. Res..

[24] Dizon L., Wiles J., Peiris-John R. (2020). What is meaningful participation for older people? An analysis of aging policies. Gerontologist.

[25] Withall J., Thompson J.L., Fox K.R., Davis M., Gray S., de Koning J., Lloyd L., Parkhurst G., Stathi A. (2018). Participant and public involvement in refining a peer-volunteering active aging intervention: Project ACE (active, connected, engaged). Gerontologist.

[26] Fernandez-Ballesteros R., Olmos R., Santacreu M., Bustillos A., Molina M.A. (2017). The role of perceived discrimination on active aging. Arch. Gerontol. Geriatr..

[27] Palmore E.B. (1968). The effects of aging on activities and attitudes. Gerontologist.

[28] Azeredo M.J., Filippin L.I., Boniatti M.M. (2024). Satisfaction with life among elderly participants in active maturity groups: A comparative study with institutionalized and community elderly individuals. Rev. DELOS.

[29] Stathi A., Withall J., Thompson J.L., Davis M.G., Gray S., de Koning J., Parkhurst G., Lloyd L., Greaves C., Laventure R. (2020). Feasibility trial evaluation of a peer volunteering active aging intervention: ACE (active, connected, engaged). Gerontologist.

[30] Zhang K., Kan C., Luo Y., Song H., Tian Z., Ding W., Xu L., Han F., Hou N. (2022). The promotion of active aging through older adult education in the context of population aging. Front. Public Health.

[31] Dogra S., Dunstan D.W., Sugiyama T., Stathi A., Gardiner P.A., Owen N. (2022). Active aging and public health: Evidence, implications, and opportunities. Annu. Rev. Public Health.

