# Supplementary material for: The Association Between Active Aging and Health-Related Quality of Life
Source: Geriatrics (Basel). 2026 Jun 17;11(3):74. doi: 10.3390/geriatrics11030074 (PMC13300381; doi:10.3390/geriatrics11030074)
Supplement: Supplementary file 1 [file geriatrics-11-00074-s001.zip › geriatrics-4251718-supplementary.pdf]

Supplementary Table S1 presents the characteristics of older adults included in and excluded from the final analytic sample. Among 3,573 eligible older adults, 2,931 were included in the final analysis and 642 were excluded because of missing information. In the excluded group, 64.2% were women, 36.9% resided in rural areas, and 61.5% were aged 75 years or older. Missing values in the excluded group were observed for education level (n = 433, 67.4%), smoking status (n = 164, 25.5%), and BMI (n = 170, 26.5%).

Supplementary Table S1. Characteristics of Included and Excluded Older Adults

Variables		Included participants	Excluded participants	Missing values in excluded older adults
		N (%)	N (%)	N (%)
Total		2931 (100)	642 (100)	
Sex				0 (0.0)
	Male	1300 (44.4)	230 (35.8)	
	Female	1631 (55.6)	412 (64.2)	
Residence				0 (0.0)
	Urban	2040 (69.6)	405 (63.1)	
	Rural	891 (30.4)	237 (36.9)	
Age group				0 (0.0)
	65-74	1810 (61.8)	247 (38.5)	
	≥75	1121 (38.2)	395 (61.5)	
Education level				433 (67.4)
	High	314 (10.7)	7 (3.3)	
	Middle	1084 (37.0)	42 (20.1)	
	Low	1533 (52.3)	160 (76.6)	
Smoke				164 (25.5)
	None	1808 (61.7)	310 (64.9)	
	Former	846 (28.9)	117 (24.5)	
	Current	277 (9.5)	51 (10.7)	
Drink				0 (0.0)
	Yes	1411 (48.1)	170 (26.5)	
	No	1520 (51.9)	472 (73.5)	
BMI				170 (26.5)
	Underweight	90 (3.1)	21 (4.4)	
	Normal	985 (33.6)	198 (42.0)	
	Overweight	785 (26.8)	105 (22.2)	
	Obesity	1071 (36.5)	148 (31.4)	

Note. Percentages for excluded older adults were calculated among participants with available data for each characteristic. Missing values in excluded older adults are presented separately. BMI, body mass index.

Supplementary Table S2 was prepared to examine the extent of ceiling effects in the HRQoL measures using the 2019 subsample, where both HINT-8 and EQ-5D were available. The mean HINT-8 and EQ-5D scores were 0.77 (SD = 0.11) and 0.89 (SD = 0.15), respectively. The ceiling proportion was 4.16% for HINT-8 and 50.83% for EQ-5D. This descriptive comparison suggests that HINT-8 was less concentrated at the maximum score than EQ-5D in the 2019 subsample.

Supplementary Figure S1 was prepared to visually compare the distribution and ceiling concentration of HINT-8 and EQ-5D scores in the 2019 subsample.
The HINT-8 scores were distributed mainly between approximately 0.60 and 0.90, with relatively limited concentration at the maximum observed score. In contrast, EQ-5D scores were heavily concentrated near 1.00, with a prominent peak at the maximum value. This pattern is consistent with the descriptive results showing a ceiling proportion of 4.16% for HINT-8 and 50.83% for EQ-5D.
Overall, the histogram indicates that EQ-5D showed a stronger ceiling effect, whereas HINT-8 showed a more dispersed distribution and lower concentration at the maximum score in the 2019 subsample.

Supplementary Table S2. Descriptive Statistics and Ceiling Proportions of HINT-8 and EQ-5D in the 2019 Subsample

Measure	N	Mean	SD	Ceiling, n (%)
HINT-8	1442	0.77	0.11	60 (4.16)
EQ-5D	1442	0.89	0.15	733 (50.83)

Note. The comparison was restricted to the 2019 subsample because both HINT-8 and EQ-5D were available in that year. Ceiling for HINT-8 was defined as the maximum observed HINT-8 score in the 2019 subsample, and ceiling for EQ-5D was defined as an EQ-5D index score of 1. HINT-8, Health-Related Quality of Life Instrument with Eight Items; EQ-5D, EuroQol five-dimension instrument; SD, standard deviation; Q1, first quartile; Q3, third quartile.

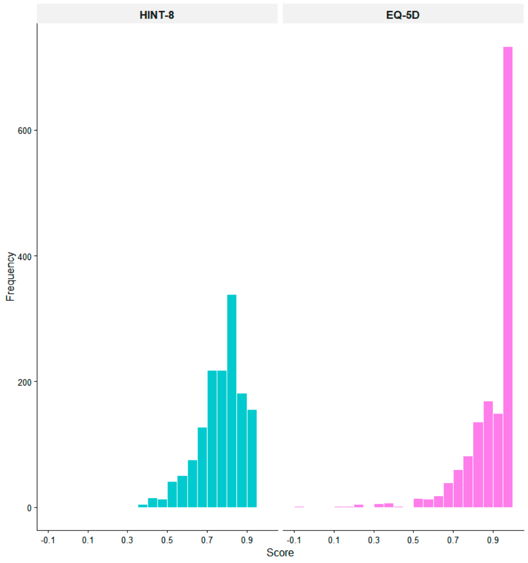

Supplementary Figure S1. Distribution of HINT-8 and EQ-5D Scores in the 2019 Subsample

Note. The histograms show the distributions of HINT-8 and EQ-5D scores among participants in the 2019 subsample, where both measures were available. HINT-8, Health-Related Quality of Life Instrument with Eight Items; EQ-5D, EuroQol five-dimension instrument.

Supplementary Table S3 presents multicollinearity diagnostics for the main and domain-specific models. Model 1 included AAI and covariates, whereas Model 2 included the three AAI domains, health, participation, and security, along with covariates. In Model 1, the VIF for AAI was 1.254, and the maximum VIF among all variables was 2.055. In Model 2, the VIF values for the health, participation, and security domains were 1.113, 1.191, and 1.345, respectively, and the maximum VIF among all variables was 2.077. All VIF values were below 5, and all tolerance values were above 0.20, indicating no substantial multicollinearity in either model.

Supplementary Table S3. Multicollinearity Diagnostics for the Main and Domain-Specific Models

Variable	Model 1		Model 2	
	VIF	Tolerance	VIF	Tolerance
AAI	1.254	0.798	-	-
Health	-	-	1.113	0.898
Participation	-	-	1.191	0.839
Security	-	-	1.345	0.744
Covariates				
Sex	2.055	0.487	2.077	0.481
Residence	1.052	0.951	1.055	0.948
Age group	1.177	0.849	1.229	0.813
Education level	1.196	0.836	1.241	0.806
Smoking	1.862	0.537	1.862	0.537
Drinking	1.174	0.852	1.178	0.849
BMI	1.009	0.991	1.011	0.989

Note. Model 1 included AAI and covariates, and Model 2 included the three AAI domains (health, participation, and security) and covariates. Tolerance was calculated as 1/VIF. AAI, Active Aging Index; BMI, body mass index; VIF, variance inflation factor.

Supplementary Table S4 presents the results of the multi-group path analysis by educational level. The association between AAI and HINT-8 was statistically significant in all educational groups. For each 0.1-unit increase in AAI, the HINT-8 score increased by 0.019 points among participants with high education (SE = 0.003, 95% CI: 0.014–0.025, $p < .0001$), 0.021 points among those with middle education (SE = 0.002, 95% CI: 0.018–0.024, $p < .0001$), and 0.026 points among those with low education (SE = 0.002, 95% CI: 0.023–0.029, $p < .0001$). The path difference test showed that the AAI-HINT-8 association differed across educational groups ($\Delta\chi^2 = 6.47$, $\Delta df = 2$, $p = .039$).

Supplementary Table S4. Multi-group path analysis of the association between AAI and HINT-8 by educational level

Educational level	β	SE	95% CI lower	95% CI upper	P value
High	0.019	0.003	0.014	0.025	<.0001
Middle	0.021	0.002	0.018	0.024	<.0001
Low	0.026	0.002	0.023	0.029	<.0001
Path difference test	$\Delta\chi^2 = 6.47$	$\Delta df = 2$	-	-	0.039

Note. β represents the unstandardized path coefficient per 0.1-unit increase in AAI. The path difference test compared the unconstrained and constrained models across educational strata. AAI, Active Aging Index; HINT-8, Health-Related Quality of Life Instrument with Eight Items; SE, standard error; CI, confidence interval; $\Delta\chi^2$, chi-square difference; Δdf, difference in degrees of freedom.
